# Efficacy of Transcranial Direct Current Stimulation Combined with Conventional Swallowing Rehabilitation Training on Post-stroke Dysphagia

**DOI:** 10.1007/s00455-023-10581-2

**Published:** 2023-05-05

**Authors:** Lingyan Wang, Aiqun Shi, Hui Xue, Qiwei Li, Jiasheng Wang, Heliang Yang, Hong Hong, Qiaomiao Lu, Jiaping Cheng

**Affiliations:** 1 Department of Rehabilitation Medicine, Jinhua Hospital of TCM Affiliated to Zhejiang University of Traditional Chinese Medicine, 496# Shuangxixi Road, 321017 Jinhua, Zhejiang China; 2 Electroencephalogram Room, Jinhua Hospital of TCM Affiliated to Zhejiang University of Traditional Chinese Medicine, 496# Shuangxixi Road, 321017, Jinhua, Zhejiang China

**Keywords:** Stroke, Dysphagia, Transcranial direct current stimulation, Rehabilitation, Long-term efficacy

## Abstract

To observe the clinical effects of transcranial direct current stimulation (tDCS) combined with conventional swallowing rehabilitation training on post-stroke dysphagia and explore its long-term efficacy. A total of 40 patients with dysphagia after the first stroke were randomly divided into a treatment group (*n* = 20) and a conventional group (*n* = 20). The treatment group received tDCS combined with conventional swallowing rehabilitation training, while the conventional group only received conventional swallowing rehabilitation training. The Standardized Swallowing Assessment (SSA) Scale and the Penetration-Aspiration Scale (PAS) were used to assess dysphagia before and after treatment, at the end of 10 treatments, and at the 3-month follow-up. The changes in infection indicators [the white blood cell (WBC), C-reactive protein (CRP) and procalcitonin (PCT)], the oxygenation indicator [arterial partial pressure of oxygen (PaO_2_)] and nutrition-related indicators [hemoglobin (Hb) and serum prealbumin (PAB)] were compared before and after treatment. The SSA and PAS scores were lower in both groups after treatment than before treatment, and the difference was statistically significant (*P* < 0.01). The SSA and PAS scores of the treatment group were lower than those of the conventional group before and after treatment and during follow-up, and the difference was statistically significant (*P* < 0.05, *P* < 0.01). A within-group comparison showed that WBC, CRP and PCT after treatment were lower than those before treatment, and the difference was statistically significant (*P* < 0.05). The PaO_2_, Hb and serum PAB were higher after treatment than before treatment, with a statistically significant difference (*P* < 0.05). The WBC, CRP and PCT of the tDCS group were lower than those of the conventional group, and PaO_2_, Hb and serum PAB were higher in the treatment group than in the conventional group, with a statistically significant difference (*P* < 0.01). The tDCS combined with conventional swallowing rehabilitation training can improve dysphagia with a better effect than conventional swallowing rehabilitation training and has a certain long-term efficacy. In addition, tDCS combined with conventional swallowing rehabilitation training can improve nutrition and oxygenation and reduce infection levels.

## Introduction

Swallowing is a set of rapid, highly coordinated neuromuscular actions that begin with lip closure and terminate with upper esophageal sphincter closure after a bolus passes through [1]. The prevalence of dysphagia ranges from 8.1 to 80% [2]. Dysphagia, which leads to dehydration, malnutrition, and recurrent aspiration pneumonia, is one of the main reasons for increased stroke mortality [3–5]. The conventional swallowing rehabilitation techniques include food intake training and indirect swallowing training, such as oral sensory stimulation, tongue muscle resistance training, pharyngeal constrictor strength training, balloon dilation technique and neuromuscular electrical stimulation therapy [6–8]. However, there are still 11–50% of stroke patients with dysphagia who develops into long-term chronic conditions due to slow recovery, which seriously affects their quality of life [3, 9]. Therefore, how to quickly and effectively restore the swallowing function of patients, reduce the risk of aspiration and improve the nutritional status is a clinical problem that needs to be urgently solved.

At present, there is no standard treatment plan for post-stroke dysphagia. With the increasing use of non-invasive brain stimulation (NIBS) technology, in addition to the sensory and motor training of peripheral swallowing muscles for the treatment of dysphagia, central regulation technology has become a hot topic in the field of swallowing rehabilitation. The NIBS, such as transcranial direct current stimulation (tDCS), can promote brain plasticity, which can improve the swallowing function of patients with post-stroke dysphagia [10–13]. In terms of improving complications related to post-stroke dysphagia, Huiwen Mao [14] found that the tDCS therapy combined with conventional training can improve patients’ swallowing function and nutritional status and reduce infection. However, most studies only reported immediate effects but did not discuss long-term efficiency [15, 16].

In our study, combined with conventional swallowing rehabilitation therapy, tDCS therapy was applied to patients with post-stroke dysphagia to observe the effect of the technique. Given the lack of literature on the long-term efficacy of tDCS in treating post-stroke dysphagia, we observed and recorded the swallowing function of patients at the 3-month follow-up. We aimed to explore a more effective and safer rehabilitation training program for post-stroke dysphagia.

## Participants and Methods

### Patients

**Fig. 1:**
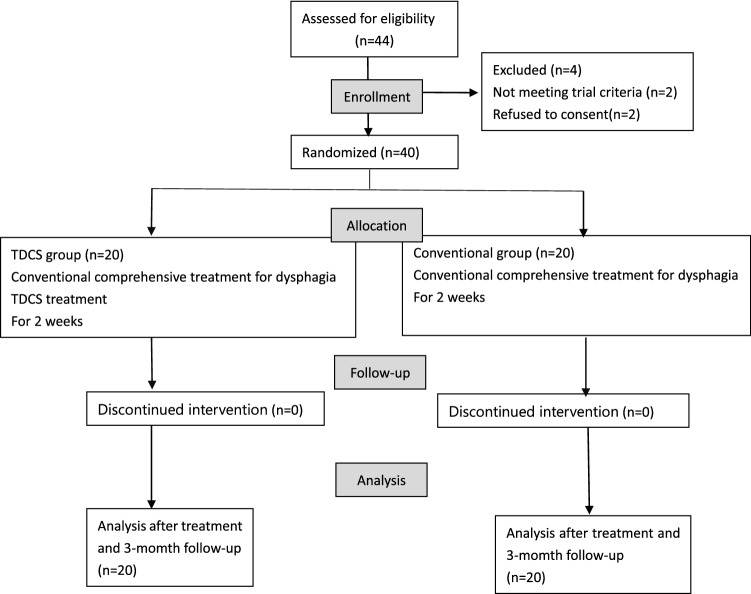
Flow diagram of participants through the trial

This parallel-group, randomized clinical trial was completed by 40 patients. From October 2019 to August 2021, 40 patients with dysphagia after the first stroke were admitted to the Department of Rehabilitation Medicine of Jinhua Hospital of Traditional Chinese Medicine affiliated to Zhejiang Chinese Medicine University. They all met the inclusion criteria and were randomly assigned to the tDCS group and the conventional treatment group, with 20 patients in each group. All patients or their families submitted written informed consent. This study has been approved by the Medical Ethics Committee of Jinhua Hospital of Traditional Chinese Medicine. In the present study, all participants were required to satisfy the following inclusion criteria: (1) The stroke was diagnosed according to the definition of stroke by the World Health Organization and confirmed by head CT or MRI. (2) Age 30–65 years old. (3) The first or a history of stroke but no previous swallowing dysfunction and the course of the disease within 3 months, with the SSA score ≥ 22 points and the PAS grade ≥ level 4. (4) The condition is in a stable stage, with stable vital signs and no serious complications in the heart, lungs or other organs. Exclusion criteria included: (1) Previous history of dysphagia. (2) Age > 65 years old or < 30 years old. (3) CT or MRI shows significant cerebral edema. (4) Patients with a history of epilepsy or continuous use of central nervous system-active drugs that can interfere with the effect of tDCS, such as carbamazepine, phenytoin and valproic acid. (5) Unstable condition and other serious heart, lung, liver and kidney disease. The enrollment flow diagram is shown in Fig. 1.

## Methods

The conventional treatment group received conventional swallowing rehabilitation training, while the tDCS group received tDCS training based on conventional swallowing rehabilitation training. The specific methods are as follows:

### Conventional Swallowing Rehabilitation Training

Targeted training was adopted according to different periods of dysphagia: (1) Breathing training: patients were instructed to perform abdominal breathing, blow out candles or paper strips after deep inhalation or whistle several times until they felt tired. (2) Ice stimulation training: iced cotton swabs were used to stimulate the buccopharyngeal part twice a day for 5 min each time. (3) Tongue muscle extension training: the tongue was pulled in all directions with tongue suction for 2 min each time. (4) Oropharyngeal stimulation with air-pulse training [17]: A 5 mm-diameter open-end silicone tube was inserted into the patient's tonsils and connected to the other end to a manually operated air pump. The therapist held the air pump to pump inwards every 5 s for 30 times. (5) Shaker exercise [18]: the patient was asked to lie supine on the bed and raise their heads to look at their toes for 30 repetitions, and their shoulders could not leave the bed surface. Patients who could not lift their heads were assisted by a therapist. (6) Food intake training [6]: Patients were asked to lie in a semi-recumbent position. They started eating a small dose of 1 ml of food, which was increased depending on their individual conditions. Food intake training was conducted once a day, 10 min each time, for 2 weeks. (7) VitalStim electrical stimulation: The neuromuscular electrical stimulator (NMES) was the American VitalStim5900 swallowing disorder treatment device (wave width: 700 ms; output intensity: 0–15 mA; frequency 30–80 Hz; production registration number 3129926). During treatment, two electrodes, A and B, in channel 1 were horizontally arranged above the hyoid bone, and two electrodes, C and D, in channel 2 were horizontally arranged above the thyroid cartilage and on both sides of the midline, respectively. The stimulation intensity was 3–10 mA, once a day for 20 min each time, for 2 weeks.

### The tDCS Protocol

**Fig. 2:**
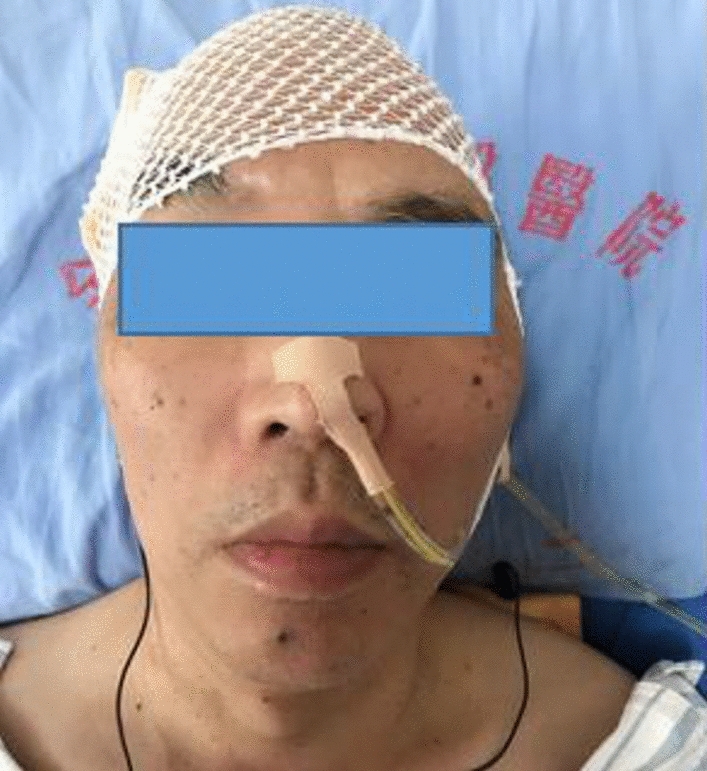
The tDCS treatment

The tDCS was delivered using a battery-driven constant-current direct current stimulator (ActivaDose® II tDCS Starter Kit, Taiwan, China) through 2 saline-soaked electrodes. The tDCS anode was placed on the swallowing sensorimotor cortex of the unaffected side. The center of a tDCS anodal electrode pad with a size of 5.0 cm × 5.0 cm was placed on the swallowing sensorimotor cortex during the operation. According to the positioning method of the International 10–20 Electrode System, the swallowing sensorimotor cortex of the left brain was located at the midpoint of C3 to T3, while that of the right brain was located at the midpoint of C3 to T3. The cathode was placed on the opposite shoulder, with 1 mA of current, 20 min each time, once a day, 5 days a week, and a total of 2 weeks (Fig. 2).

## Outcome Measurement

### Standardized Swallowing Assessment (SSA) Scale [19]

The SSA was performed in three parts: (1) Clinical examination, including consciousness, head and trunk control, breathing, lip closure, soft palate movement, laryngeal function, gag reflex and spontaneous cough. The score was 8–23 points. (2) Patients were instructed to swallow 5 ml of water three times to observe the laryngeal movement and wheeze during swallowing. The score was 5–11 points. (3) If there was no abnormality in the above examination, the patient was instructed to swallow 60 ml of purified water to observe swallowing time and cough. The score was 5–12 points. The total score ranged between 18 and 46 points. Higher scores indicated a worse swallowing function.

### Penetration-Aspiration Scale (PAS)

The PAS is an 8-point scale used to characterize the location of airway invasion events and patients’ responses during swallowing [20]: score 1, matter does not enter the airway; score 2, matter enters the airway, remains above the level of the vocal folds, and is ejected from the airway; score 3, matter enters the airway, remains above the level of the vocal folds, but is not ejected from the airway; score 4, matter enters the airway, contacts the vocal folds, and is ejected from the airway; score 5, matter enters the airway, contacts the vocal folds, but is not ejected from the airway; score 6, matter enters the airway, passes below the level of the vocal folds, and is ejected into the larynx or out of the airway; score 7, matter enters the airway, passes below the level of the vocal folds, but is not ejected out of the trachea despite force; score 8, matter enters the airway, passes below the level of the vocal folds, and ejects without force. The PAS was assessed under the Video-fluoroscopic Swallowing Study (VFSS) or the bedside Fiberoptic Endoscopic Evaluation of Swallowing (FEES).

### Blood Indicators

Some blood indicators were used to assess dysphagia before and after treatment and at the 3-month follow-up. Infection indicators, including the white blood cell (WBC), C-reactive protein (CRP), and procalcitonin (PCT), were used to evaluate infection. The oxygenation indicator, arterial partial pressure of oxygen (PaO_2_), was drawn in the absence of oxygen and was recorded as oxygenation capacity. Nutrition indicators, such as hemoglobin (Hb) and serum prealbumin (PAB), were recorded as nutritional status.

### Time of Assessment

The SSA, PAS, infection indicators, oxygenation indicator and nutritional indicators were evaluated before and after treatment, and SSA and PAS were reassessed at the 3-month follow-up.

### Statistical Analysis

All data were analyzed by SPSS 22.0 software (IBM, Inc.) and were expressed as mean and standard deviation (SD) or numbers with proportions. The baseline demographic was compared between the tDCS and conventional treatment groups using the Mann–Whitney U test or Chi-square test. Then, SSA, PAS, WBC, PCT, CRP, PAB, and Hb between the two groups before and after treatment were compared by an independent sample t-test and paired t-test within each group or Mann–Whitney U test. If the data conformed to a normal distribution, the t-test was used, and if not, the Mann–Whitney U test was used. *P* value < 0.05 was considered statistically significant.

## Results

### Baseline Characteristics

There were 12 males and 8 females in the tDCS group, with an average age of 52.7 ± 8.0 years old. The disease course was 50.65 ± 21.82 days. Additionally, 11 patients had a left-side stroke, and the remaining 9 had a right-side stroke; 13 patients had an intracerebral hemorrhage, and 7 cases patients had cerebral infarction. The NIHSS score was 12.4 ± 2.74, and the MMSE score was 16.55 ± 3.61, with the risk factors for underlying diseases of 1.45 ± 0.51. In the conventional treatment group, there were 9 males and 11 females. The average age was 51.65 ± 9.74 years old, and the disease course was 50.45 ± 17.48 days. In addition, 12 patients had a left-side stroke, and the remaining 8 had a right-side stroke; 14 patients had an intracerebral hemorrhage, and 6 patients had cerebral infarction. The NIHSS score was 11.9 ± 3.29, and the MMSE score was 16.0 ± 4.37, with the risk factors for underlying diseases of 1.4 ± 0.6. The baseline data of the two groups had no significant difference (*X*^2^ > 0.05, *P* > 0.05) (Table 1).

**Table 1:** Baseline characteristics of subjects. (Mean ± SD)

Group	*n*	Gender		Age (year)	Course	Lesion		Type		Risk factor	NIHSS	MMSE
		Male	Female			Left	Right	Hemorrhage	Infarction			
tDCS	20	12	8	52.7 ± 8.0	50.65 ± 21.82	11	9	13	7	1.45 ± 0.51	12.4 ± 2.74	16.55 ± 3.61
conventional	20	9	11	51.65 ± 9.74	50.45 ± 17.48	12	8	14	6	1.4 ± 0.6	11.9 ± 3.29	16.0 ± 4.37
X^2^		0.34				0.749		0.736				
*P* value				0.799	0.947					0.678	0.565	0.583

*tDCS* transcranial direct current stimulation; *SD* standard deviation. Risk factors include hypertension, diabetes mellitus, atrial fibrillation, intracranial arteriovenous malformation, hyperlipidemia, and hyperhomocysteinemia, each of which is recorded as one score. *NIHSS* National Institutes of Health Neurological Impairment Score. *MMSE* Mini-mental State Examination

### Clinical Baseline Information of Participants Before and After Treatment

In terms of swallowing function, SSA and PAS scores were 35.4 ± 2.7 and 5.3 ± 1.08 in the tDCS group and 34.70 ± 2.51 and 5.25 ± 0.91 in the conventional treatment group, respectively. There was no significant difference between the two groups (^a^*P* > 0.05)(Table 2). Regarding infection indicators, the tDCS and conventional treatment groups showed 9.44 ± 2.07 and 10.14 ± 1.78 for WBS, 18.9 ± 20.62 and 17.73 ± 18.16 for CRP, and 0.31 ± 0.32 and 0.44 ± 0.36 for PCT. In terms of the oxygenation indicator, PaO_2_ is 67.27 ± 3.36 and 66.89 ± 3.51 in the tDCS and conventional treatment groups. For nutritional indicators, the tDCS and conventional comprehensive treatment groups showed 110.75 ± 9.8 and 110.25 ± 10.0 for Hb and 245.75 ± 68.64 and 223.10 ± 62.04 for PAB. There was no significant difference between the two groups (^a^*P* > 0.05) (Table 3).

**Table 2:** Parameters of the two groups before and after the treatment and at the 3-momth follow-up (Mean ± SD)

Group (*n* = 40)	SSA				PAS		
	Before	After	3-month		Before	After	3-month
tDCS (*n* = 20)	35.40 ± 2.7	27.3 ± 3.46	22.25 ± 1.97		5.3 ± 1.08	2.95 ± 1.05	1.95 ± 0.94
Conventional (*n* = 20)	34.70 ± 2.51	29.8 ± 2.67	24.25 ± 3.46		5.25 ± 0.91	4.1 ± 1.02	2.75 ± 0.55
*P* value (Baseline)	0.402^a^				0.925^a^		
*P* value (tDCS)		0.000^b^				0.000^b^	
*P* value (Conventional)		0.000^b^				0.000^b^	
*P* value (Comparison)		0.015^c^	0.002^d^			0.002^c^	0.003^d^

*SSA* Standardized Swallowing Assessment. *PAS* Penetration Aspiration Scale. ^a^*P* > 0.05, ^b^*P* < 0.01, ^c^*P* < 0.05, ^c^*P* < 0.01, ^d^*P* < 0.01

^*a*^*P*, *P* values for baseline comparisons

^b^*P*, *P* values for within-group comparisons before and after treatment

^c^*P*, *P* values for comparison between the two groups after treatment

^d^*P*, *P* values for comparison between the two groups at the 3-month follow-up

**Table 3:** The clinical indicators of the two groups (mean ± SD)

Group (*n* = 40)	Time	WBC (10^9^/L)	CRP (mg/L)	PCT (ng·ml)	PaO_2_ (mmHg)	Hb (g/L)	PAB (g/L)
tDCS	Before	9.44 ± 2.07ab	18.9 ± 20.62ab	0.31 ± 0.32ab	67.27 ± 3.36ab	110.75 ± 9.8ab	245.75 ± 68.64ab
(*n* = 20)	After	8.18 ± 1.89c	4.23 ± 6.5c	0.12 ± 0.17c	73.9 ± 4.34c	117.05 ± 10.35c	292.95 ± 75.69c
Conventional	Before	10.14 ± 1.78	17.73 ± 18.16	0.44 ± 0.36	66.89 ± 3.51	110.25 ± 10.0	223.10 ± 62.04
(*n* = 20)	After	9.35 ± 1.55c	12.81 ± 15.79c	0.27 ± 0.26c	71.12 ± 2.87c	111.0 ± 8.36c	245.95 ± 58.07c
*P* value (Baseline)		0.257^a^	0.849^a^	0.212^a^	0.729^a^	0.874^a^	0.281^a^
After treatment							
*P* value (tDCS)		0.000^b^	0.009^b^	0.007^b^	0.000^b^	0.000^b^	0.000^b^
*P* value (Conventional)		0.000^b^	0.002^b^	0.000^b^	0.000^b^	0.000^b^	0.000^b^
*P* value (Comparison)		0.039^c^	0.034^c^	0.033^c^	0.021^c^	0.049^c^	0.034^c^

*WBC* white blood cell. *CRP* C-reactive protein. *PCT* procalcitonin, *PaO*_*2*_ arterial partial pressure of oxygen. *Hb* hemoglobin. *PAB* blood serum prealbumin

^a^*P* > 0.05, ^b^*P* < 0.01, ^c^*P* < 0.05

### Comparison of the Effects of the Two Groups

#### Comparison of Swallowing Function Results Before and After Treatment in the tDCS Group

The SSA score and the PAS score after tDCS treatment were 27.3 ± 3.46 and 2.95 ± 0.15, both of which were improved compared with those before the treatment (^c^*p* < 0.05, ^d^*P* < 0.01) (Table 2). In terms of infection indicators, WBS, CRP and PCT were 8.18 ± 1.89, 4.23 ± 6.5 and 0.12 ± 0.17 after the treatment. The oxygenation indicator, PaO_2_, was 73.9 ± 4.34 after the treatment. For nutritional indicators, Hb was 117.05 ± 10.35, and PAB was 292.95 ± 75.69 after the treatment, both of which were improved compared with those before the treatment (^b^*P* < 0.01) (Table 3).

#### Comparison of Swallowing Function Results Before and After Treatment in the Conventional Group

The SSA score and the PAS score after the treatment were 29.8±2.67 and 4.1±1.02, both of which were improved compared with those before the treatment (^b^*p*<0.01) (Table 2). Regarding infection indicators, WBS, CRP and PCT were 8.18±1.89, 12.81±15.79, and 0.27±0.26 after the treatment. The oxygenation indicator, PaO_2_, was 71.12±2.87 after treatment. For nutritional indicators, Hb and PAB were 111.0±8.36 and 245.95±58.07 after the treatment, both of which were improved compared with those before the treatment (^b^*P*<0.01) (Table 3).

#### Comparison Between the Two Groups After Treatment and at the 3-Month Follow-Up

In terms of swallowing function, SSA and PAS scores in the tDCS group were lower than those in the conventional group after treatment and at the 3-month follow-up. (^c^*P*<0.05, ^c^*P*<0.01, ^d^*P*<0.01). For infection, oxygenation and nutritional indicators, the tDCS group was improved compared with the conventional group after treatment (^c^*p*<0.05) (Table 2).

## Discussion

Swallowing involves a series of processes, such as cognitive processing, coordination of swallowing muscles and pleasure in eating, and the brain produces complex network connections. There is no uniform standard for tDCS treatment of dysphagia, such as the stimulation site, current, stimulation duration, electrode size, and treatment frequency. The tDCS is a non-invasive tool for inducing changes in cortical excitability via two electrodes, where the anode increases cortical excitability by depolarizing the resting membrane potential, while the cathode hyperpolarizes the resting membrane potential to reduce cortical excitability and promote the remodeling of brain functions [16]. Many studies have shown that tDCS is a central stimulation strategy that has promising results in treating post-stroke dysphagia [21–23]. However, a consensus has not yet been reached for parameters such as current intensity, session duration, treatment period, anode design and cathode design. Most importantly, the optimal stimulation protocol remains uncertain [21].

### Stimulation Design

According to the stimulation design, in most studies, the anode was placed in the contralateral brain since it is less affected by neuronal loss and tissue damage and responds more consistently to tDCS stimulation. Kumar et al. [12] found that repeated application of anodal tDCS to the unaffected swallowing cortex combined with timed effortful swallowing was associated with significant swallowing improvement over the sham group. However, Yang Eun Joo et al. [24] showed that after anode stimulation of the affected side of the brain after 10 days’ intervention, Functional Dysphagia Scale (FDS) scores were improved in both the tDCS and sham groups without significant differences. However, after 3 months of intervention, anodal tDCS showed greater improvement in FDS compared to the sham group. In a positron emission tomography (PET) study, patients who received anodal tDCS showed hypermetabolic spots, but there was no difference in the unaffected hemisphere after sham stimulation. This result suggested that tDCS may activate large cortical areas related to the recovery of swallowing function, not just a single area. Therefore, the role of the affected side of the brain in the recovery of swallowing function in stroke patients cannot be ignored [24]. Yang Seung Nam et al. [11] showed that there was no difference in the recovery of swallowing function, whether it is stimulated on the unaffected side or on the affected side. However, Lin Qian et al. [25] showed that tDCS might be effective for the recovery of dysphagia in post-stroke patients, both in the acute and chronic phases. Additionally, the effect of anodal tDCS on unaffected hemispheres is larger. However, there are many heterogeneities in patients and efficacy evaluation methods included in the literature. Further large-sample multi-center clinical studies are needed. Li Y et al. [26] found that both unilateral and bilateral hemispheric anodal tDCS combined with conventional therapies are helpful for the recovery of swallowing function in patients with chronic dysphagia induced by stroke, with bilateral anodal tDCS showing more significant improvement.

### Stimulation Dose

There is no uniform standard for the stimulation intensity and duration of tDCS. Kumar Sandeep et al. [12] showed that the anode of tDCS was placed in the unaffected side of the brain; the current was 2 mA and stimulated the brain 30 min per day for 5 days, which contributed to swallowing rehabilitation after stroke. After summarizing nine studies on the therapeutic effect of tDCS on post-stroke dysphagia, Ayodele Sasegbon [21] found that a single tDCS lasted for 20–30 min and was repeated 4–16 times. He Kelin [27] analyzed 15 trials of tDCS for the treatment of post-stroke dysphagia and found that the maximum current intensity was 2 mA, the minimum current intensity was 1 mA, and the intervention duration was from 5 days to 2 months. The stimulation with a short duration is effective, possibly because tDCS alters the plasticity of neurons in the swallowing motor cortex, which is related to the long-term potentiation (LTP) effect of tDCS in the postsynaptic membrane [28], and this alteration is immediate and stable. The efficacy of tDCS for post-stroke dysphagia could be improved when stimulation intensity was 1–2 mA [29]. Jefferson [30] believed that higher intensity (1.5 mA) or longer duration (20 min) could contribute to stronger stimulation to the pharyngeal cortex. Feng WW [31] showed that current with an intensity equal to or larger than 2 mA could not be easily tolerated by normal people or stroke patients and might lead to dangers; a high stimulation intensity does not necessarily mean a significant effect on cerebral cortical excitability. In this study, we stimulated the contralateral sensorimotor cortex area of the post-stroke patients for 20 min, with the current intensity being 1 mA. The results showed that the stimulation contributed to the swallowing rehabilitation of stroke patients, which was similar to the findings in most previous studies. The effect of tDCS on post-stroke dysphagia, the course of treatment and the stimulation intensity need further study.

### Short and Long-Term Efficacy

In the 3-month follow-up, we found that the swallowing function of patients in the tDCS treatment group was significantly better than that of patients in the conventional treatment group, indicating that tDCS has a certain long-term efficacy against dysphagia. However, how long this therapeutic efficacy will last remains unclear. That is, whether tDCS can reverse the damage of the swallowing functional connection pathway and whether its efficacy has long-term stability need to be further studied. The completion of swallowing movement requires the normal coordination of the cerebral cortex and subcortical areas, cerebellum, peripheral nerves and related muscles and sensory input. Swallowing is regulated by bilateral brains, but bilateral regulation is not absolutely symmetrical [32]. The brainstem swallowing center has bilateral cortical innervation, and measures to enhance cortical input and sensorimotor control of brainstem swallowing may benefit recovery from dysphagia [12]. Song Myeongseop [33] recorded EEGs during direct current stimulation and during a 5-min resting state before and after the stimulation and found that tDCS of the left dorsolateral prefrontal cortex made the brain into a ready state for efficient cognitive functioning by increasing the beta-frequency power. Yuan Ying [16] performed three weeks of anodal tDCS over the bilateral primary sensorimotor cortex of swallowing and conventional treatments and found that anodal tDCS might be an effective treatment protocol for swallowing apraxia, and the recovery from dysphagia could be related to increased excitability of the swallowing cortex. Sánchez-Kuhn Ana [34] performed tDCS on a patient with severe dysphagia after left cerebellar infarction by anodal stimulation on the left M1 area (1 mA, 20 min) and found that the white matter fiber connections in the left cerebellar peduncles increased, and bilateral symmetry gradually appeared. Sánchez-Kuhn Ana was the first to attempt to assess the potential connectivity changes brought by tDCS and swallowing training on a post-stroke dysphagia patient. These studies seem to explain why tDCS has long-term benefits, but the functional and microstructural changes need to be further investigated, and electrophysiological studies that explain these long-lasting behavioral improvements are required.

### Study Limitations

The limitations of this study are as follows. First, only 40 patients were included, and their disease course was within 3 months. Patients with chronic swallowing disorders were not included. The small sample size makes it hard to draw general conclusions. Second, the follow-up time was 3 months, which was not sufficient for the analysis of the microstructure of the brain to corroborate the findings. Third, in this study, we did not set a sham tDCS stimulation group to ensure the reliability of the study results. Fourth, the SSA/PSA assessors were not blinded during the study, which may have affected the study results.

## Conclusion

In this randomized clinical trial study, we investigated the effect of the stimulation to the unaffected swallowing sensorimotor cortex by tDCS combined with conventional swallowing rehabilitation training on post-stroke dysphagia. It was found that tDCS combined with conventional swallowing rehabilitation training improved the swallowing ability and nutritional state of patients both after 10 times of treatments and the 3-month follow-up. In addition, tDCS combined with conventional swallowing rehabilitation could improve oxygenation and reduce infection levels. This study preliminarily confirmed that tDCS combined with conventional swallowing rehabilitation could relieve dysphagia and has a certain long-term efficacy better than that of single conventional swallowing rehabilitation. This study provides a reference for the introduction of the combination of tDCS and conventional swallowing rehabilitation into clinical practice, and further studies are warranted to assess the clinical utility of this novel combination therapy in dysphagic patients.

In the future, further high-quality research will be conducted, and brain neuroimaging, electroencephalogram analysis, evoked potentials, functional near-infrared spectroscopy, magnetoencephalography, functional magnetic resonance imaging, diffusion tensor imaging and other methods will be applied for accurate positioning and efficacy evaluation of the efficacy of tDCS for dysphagia treatment.
